# Efficacy of Lidocaine Spray for Pain Reduction during Colposcopy-Directed Cervical Biopsies: A Randomized Controlled Trial

**DOI:** 10.3390/medicina60040630

**Published:** 2024-04-13

**Authors:** Ornwitsanate Mongkolmafai, Dhammapoj Jeerakornpassawat, Charuwan Tantipalakorn, Kittipat Charoenkwan, Prapaporn Suprasert, Jatupol Srisomboon, Theera Tongsong

**Affiliations:** Department of Obstetrics and Gynecology, Faculty of Medicine, Chiang Mai University, Chiang Mai 50200, Thailand; anotha@hotmail.com (O.M.); dhammapoj.zhou@gmail.com (D.J.); kittipat.c@cmu.ac.th (K.C.); prapaporn.su@cmu.ac.th (P.S.); jatupol1957@hotmail.com (J.S.); theera.t@cmu.ac.th (T.T.)

**Keywords:** cervical biopsy, colposcopy, lidocaine, pain relief

## Abstract

*Objectives*: The objective of this study was to evaluate the efficacy of lidocaine spray in reducing the pain during colposcopy-directed cervical biopsy (CDB). *Methods*: From December 2017 to February 2019, 312 women undergoing CDBs were enrolled. The participants were randomized to three groups: group 1 (lidocaine spray), in which lidocaine spray was applied thoroughly to the cervix; group 2 (placebo), in which normal saline was applied thoroughly to the cervix; and group 3 (control), in which no anesthetic agent was applied to the cervix. Each woman completed a 10 cm visual analog scale to classify the subjective pain experience at three time points: baseline, immediately after biopsy, and 10 min after the procedure. The primary outcome of this study was the biopsy pain score. *Results*: The 312 enrolled women were randomly assigned to the three groups, amounting to 104 women per group. The clinical and pathological characteristics of the participants in all groups were comparable. The baseline, the biopsy, and the post-procedure pain scores were comparable among the three groups. There was a significant increase in the pain score from baseline to biopsy and from baseline to post-procedure in each group. The pain-score changes from baseline to biopsy in the lidocaine spray group significantly decreased when compared with the normal saline group (<0.001), and tended to decrease, though not significantly (*p* = 0.06), when compared with the control group. No complication with the intervention was observed. *Conclusions*: The application of lidocaine spray to the cervix has the benefit of reducing the pain associated with CDBs by a small amount. However, the intervention is safe and may be considered in nulliparous and/or overly anxious women undergoing the procedure.

## 1. Introduction

A colposcopy is the cornerstone of cervical cancer prevention. It plays an important role in decreasing the incidence and mortality of cervical cancer. A colposcopy-directed cervical biopsy (CDB) is a minimally invasive outpatient diagnostic procedure performed in women with an abnormal cervical cytology or positive human papillomavirus (HPV) testing [1]. However, without anesthesia, the procedure can be associated with different degrees of suffering and pain. Recently, the American Society for Colposcopy and Cervical Pathology (ASCCP) recommended taking at least two and up to four biopsies of the abnormal lesions detected during colposcopy [2]. Previous reports noted that taking a single biopsy from the cervix may miss up to 40% of precancerous cervical lesions [3,4,5]. Thus, the currently recommended practice may cause the patients more discomfort, a burning sensation, and pain associated with the biopsy. During the examination, additional interventions may be performed, including endocervical brushing (ECB), endocervical curettage (ECC), endocervical polypectomy, and an endometrial aspiration biopsy, for further evaluation.

Local anesthesia is not routinely used for directed colposcopic cervical biopsies since the injection of anesthetic is probably as painful as the biopsy. Additionally, injection of an anesthetic may also disrupt the epithelium, making visualization of the lesion more difficult. However, though a minor procedure, a CDB performed without the use of any forms of anesthesia can cause substantial pain, as often encountered in actual practice, and such pain is of concern for many anxious patients. Therefore, several studies have been conducted to examine the effects of different anesthetic and analgesic approaches on pain relief during CDB [6,7,8,9]. The use of forced coughing, topical anesthetic gel and oral analgesic drugs appeared to be ineffective in relieving pain during the cervical biopsy procedure. A previous study reported that intracervical submucosal injection of lidocaine was effective in reducing the pain severity [8]. However, the injection itself may cause pain and discomfort. The effectiveness of lidocaine spray in pain reduction has been shown in various obstetrical and gynecological procedures, including endometrial sampling, hysteroscopy, insertion of intrauterine devices (IUDs), and loop electrosurgical excision procedures [10,11,12]. Clinical use of anesthetic and analgesic agents for CDBs is sporadic and without established evidence-based guidelines. Based on our previous study, lidocaine spray reduces pain during a colposcopy-directed cervical biopsy; however, the clinically meaningful effect of such a procedure cannot be demonstrated. In that study, the majority of participants had only one biopsy taken [13], whereas in current practice, we typically perform CDBs with two or more biopsies, causing more discomfort, as mentioned earlier. Accordingly, while lidocaine spray is attractive because of no risk and is a less invasive method without interfering with colposcopic visualization, the effectiveness of lidocaine spray has never thoroughly studied in relieving the pain associated with CDBs, especially in the cases of two or more biopsies. We hypothesized that lidocaine spray application during colposcopy could significantly reduce the pain associated with CDBs. Therefore, we conducted this study to investigate whether topical lidocaine spray application is effective in relieving pain from colposcopy-directed cervical biopsies when two or more biopsies are taken from the cervix. Also, a placebo group was added to the comparison.

## 2. Patients and Methods

This randomized controlled trial was conducted at Chiang Mai University Hospital, Thailand. The study was ethically approved by the Institutional Review Board, Faculty of Medicine, Chiang Mai University, Thailand. Women with abnormal cervical screening results and abnormal colposcopic findings that required a CDB from December 2017 to February 2019 were invited to participate in the study. All participants provided written informed consent. The study strictly followed the CONSORT criteria for randomized trials in terms of the trial design, participants (eligibility criteria, settings and locations where the data were collected), interventions, outcomes, sample size, randomization, blinding, and statistical methods, as described below. The study population was patients attending our colposcopic clinic, Chiang Mai University Hospital, Thailand. Inclusion criteria are as follows: (1) age of 15–50 years and (2) abnormal cytology screening (Pap smear) and abnormal colposcopic findings requiring CDBs. Note that an abnormal Pap smear was reported based on the Bethesda 2001 system [14] and colposcopic examination followed the guidelines recommended by American Society for Colposcopy and Cervical Pathology (ASCCP) [15]. The indications for cervical colposcopy were based on abnormal results of cervical cytology, such as ASC-H, LSIL, HSIL or ASCUS. Typically, a colposcope was used to examine the entire surface of the cervix, but the most emphasis was put on examining the squamocolumnar junction (SCJ) and transformation zone. The cervical biopsy was usually performed at the sites of abnormal colposcopic findings such as dense acetowhite, punctations, mosaicism or atypical vessels, representing sites of significant disease (cervical intraepithelial neoplasia 2, 3 or even invasive carcinoma). At least two and up to four cervical biopsies were performed under colposcopic examination, using long biopsy instruments that are able to reach the cervix to obtain the tissue of approximately 1–2 mm for each biopsy. The biopsies were usually performed on any part of the most abnormal-appearing area or from more than one abnormal-appearing area. Exclusion criteria include history of hypersensitivity to lidocaine or to other components of the solution, pregnancy, history of prior hysterectomy, bleeding disorders, drug abuse, cervical or vaginal infection, and inability to communicate in Thai. After enrollment, demographic and clinical data were prospectively collected, including age, parity, menopausal status, history of dysmenorrhea, history of dyspareunia, participant-reported anxiety, history of pelvic inflammatory disease (PID), human immunodeficiency virus (HIV) status, cervical cytology results, human papilloma virus (HPV) testing results, and final histology results.

After the participants were recruited into the study, they were randomly assigned into three groups according to a computer-generated, block of six, random allocation sequence, with a ratio of 1:1:1. Sequentially numbered, sealed, opaque envelopes were employed to provide allocation concealment. The three groups included the lidocaine spray group (Group 1), the normal saline solution group (Group 2) and the control group (Group 3). The envelopes containing treatment allocation were prepared by a research assistant who was not directly involved in this study, and were opened by project assistants. Both the participants and the project assistants were blinded to the assigned allocation. All colposcopic procedures were performed by staff members or fellows of gynecologic oncology using the same conventional technique. In Group 1 (lidocaine spray group), 4 puffs of 10% lidocaine spray (40 mg; 10 mg per puff) were applied thoroughly to the cervix. Two minutes after lidocaine application, the cervical punch biopsy was performed. For Group 2 (normal saline solution, NSS, group), 4 puffs of NSS were applied thoroughly to the cervix. Two minutes after NSS application, the cervical punch biopsy was performed. Group 3 or the control group received no lidocaine or NSS. The other steps of the procedure were identical among the three groups. In each case, the patient was placed in the lithotomy position, and the examined area was cleaned. A sterile bivalve speculum was used to inspect the cervix and the vagina, which was soaked with 5% acetic acid solution. The cervical biopsy was performed using punch biopsy forceps. Additional procedures, including ECB, ECC, endometrial aspiration biopsy or cervical polypectomy, were performed as indicated at the discretion of the attending colposcopists. Following the biopsy, hemostasis was generally achieved by the local application of the hemostatic agent. After completing the procedure, the participants were observed for 30 min before being discharged. In each case, the project assistants asked the patient to rate their pain, based on a 10 cm visual analog scale (VAS), at different points during the procedure. The pain scores immediately after speculum insertion (baseline pain score), immediately after cervical biopsy (biopsy pain score) and 10 min after the procedure (post-procedure pain score) were recorded. Patients were informed that a score of zero represented “no pain” and a score of 10 represented “unbearable pain”. The primary outcome of this study was the biopsy pain score. The number of cervical punch biopsies and the additional procedures were also recorded. Demographic and clinical data were obtained from medical records.

A 1 cm difference in the VAS between any two groups was defined as the smallest effect that was considered as clinical significance. This study needed a sample size of at least 312 cases to gain a power of 80% at a 95% confidence interval for the detection of the differences in biopsy pain scores among the 3 groups. Chi-square or Fisher’s exact test was used for categorical variables, as appropriate. A Kruskal–Wallis test with correction by Dunn’s multiple comparison test was used for a comparison of continuous variables among the three groups. A Mann–Whitney U test was employed for hypothesis testing of the pain scores between any two groups (lidocaine spray vs. NSS group, lidocaine spray vs. control groups, and NSS vs. and control group). A *p*-value of <0.05 was considered statistically significant. All statistical procedures were performed using Stata statistical software version 15 (StataCorp., College Station, TX, USA).

## 3. Results

**Figure 1:**
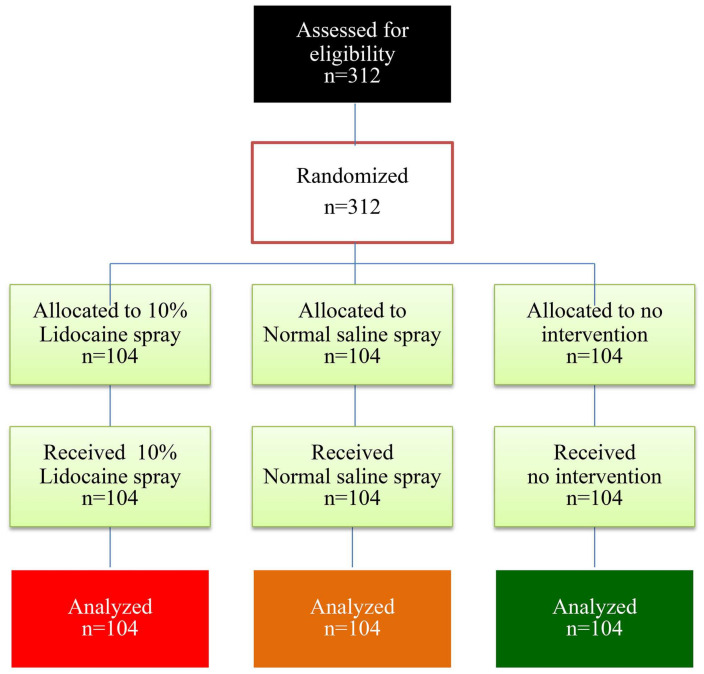
Consort flow diagram for participants involved in the trial.

Of the 312 women, 104 were randomly assigned to the lidocaine spray group, 104 to the normal saline solution spray group and 104 to the control group (no-intervention). There were no dropouts in this study, as presented in Figure 1.

The participants in the three groups were similar in terms of age, parity, history of dysmenorrhea, history of dyspareunia, menopausal status, anxiety, history of PID, HIV status, cytology results, and HPV testing results. Atypical squamous cells of undetermined significance (ASC-USs) and low-grade squamous intraepithelial lesions (LSILs) were the most common presenting abnormal cervical cytology results among the three groups. In each group, more than half of the final histology results indicated normal cervical epithelium and chronic cervicitis. A low-grade squamous intraepithelial lesion was documented in approximately one-third of the participants (Table 1).

Mostly, the procedure-related characteristics were comparable among the three groups. Only the median duration of colposcopy was significantly longer in the lidocaine spray group. Approximately half of the participants had a type-1 cervical transformation zone. The most common colposcopic impression was a low-grade intraepithelial lesion. Approximately 80% of the participants in each group underwent CDBs without additional procedures. The median number of cervical biopsies was two for all groups (Table 2).

**Figure 2:**
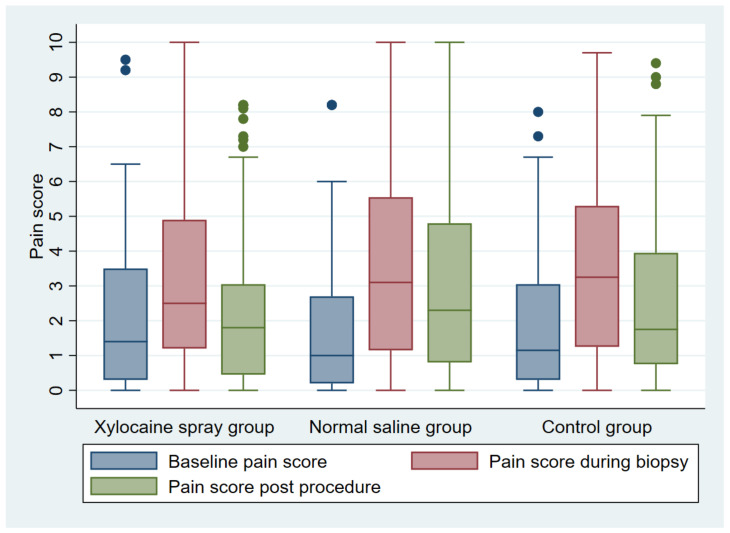
Baseline, biopsy, and post-procedure pain scores of the three study groups.

Table 3 and Figure 2 demonstrate the pain scores at various stages of the procedure. The baseline, the biopsy, and the post-procedure pain scores were comparable among the three groups. In each group, there was a significant increase in the pain score from baseline to biopsy and from baseline to post-procedure. However, when the lidocaine spray group was compared directly to the control group, there was no statistically significant difference in the pain-score changes from baseline to biopsy (*p* = 0.06) and from baseline to post-procedure (*p* = 0.29) (Table 4). No complication occurred in any of the participants.

Based on a multivariable analysis taking into account, age, parity, menopausal status, history of dysmenorrhea, history of dyspareunia, participant report of anxiety, history of PID, HIV status, cervical cytology result, HPV status, and histology result, nulliparity (adjusted β 1.15, 95% confidence interval [CI] 0.48 to 1.82, *p* < 0.001) and participants report of anxiety (adjusted β 0.94, 95% CI 0.38 to 1.50, *p* < 0.001) were significantly associated with an increased biopsy pain score. HIV seropositivity was significantly associated with a decreased biopsy pain score (adjusted β −1.04, 95% CI −1.91 to −0.17, *p* = 0.02).

## 4. Discussion

This study demonstrated a trend of a beneficial effect for women who received lidocaine spray compared to the control in lowering biopsy the change in pain score from baseline. The comparable time-point pain scores and changes in biopsy pain scores from baseline between the normal saline group and the control group imply that the placebo effect had no significant interfering role on the outcome of the assessment, and any pain-lowering effect observed essentially resulted from lidocaine spray. However, we could not demonstrate either a clinically or statistically significant benefit of lidocaine spray during CDB. Also, we noted comparable pain scores at every time point measured, including baseline, biopsy, and post-procedure, among all study groups.

Note that the biopsy pain scores were significantly lower in the lidocaine spray group compared to the normal saline group, and tended to be lower, though not significantly, when compared to the control group, while those in normal saline group and control group were comparable. These findings imply that the effects of lidocaine from the two comparisons are in agreement, suggesting that the non-significant pain reduction when compared to the control group likely occurs because the sample size is too small to express the significant small effect.

Despite being a minimally invasive procedure, CDBs are still painful in a considerable proportion of patients. Pain sensation in the cervix is transmitted to the brain via the pelvic splanchnic nerves running through the uterosacral ligaments. All types of lidocaine preparations stabilize the neuronal membrane by inhibiting ionic flow and preventing initiation and conduction of impulses [11]. Several studies reported that the CDB procedure was associated with various degrees of pain, ranging from mild to severe [16,17,18]. It should also be noted that pain associated with the entire colposcopy procedure can arise at various steps, including speculum insertion (dull discomfort), acetic acid application (burning, tingling sensation), CDBs (cramping) and additional procedures, such as endocervical biopsies, endocervical curettage, endometrial aspiration biopsies, and polypectomy. Four key themes emerged as important aspects of the overall sensory experience, i.e., levels of pain, treatment-specific sensations, anesthetic-specific sensations, and solution-specific sensations [19]. Recently, the ASCCP recommended that at least two and up to four cervical punch biopsies should be taken from the abnormal areas found during the colposcopic examination [2]. From all these considerations, the clinicians should be aware of the usually unrealized suffering and pain occurring in women undergoing the colposcopic procedure.

The effectiveness of lidocaine spray in reducing pain has been shown in other gynecological procedures, such as intrauterine device (IUD) insertion, endometrial sampling, and the loop electrosurgical excision procedure (LEEP) [11,20,21]. A meta-analysis showed that lidocaine spray was an effective medication for reducing pain during and after endometrial sampling [10]. These studies used a similar dose of lidocaine spray to our study. On the other hand, some studies could not demonstrate the effectiveness of lidocaine spray or lidocaine gel in some procedures, such as endometrial sampling and IUD insertion [22,23]. These conflicting results may result from differences in dosage, duration of administration and types of procedures. Additionally, the sample size in some studies was too small to show the small but significant effects of lidocaine spray.

Oyama et al. [8] compared the pain scores during CDBs between women who received 1% lidocaine injection and those with no injection before the biopsy procedure. The injection of lidocaine resulted in a reduction in the pain scores from cervical biopsies (4.0 to 1.1, *p* < 0.001). However, submucosal injection of local anesthetics frequently gives rise to bleeding and may interfere with the colposcopic visualization required for proper biopsy of suspected lesions. In our experience, the anesthetic injection itself is associated with significant pain and discomfort. The use of topical anesthetic spray would potentially solve this problem. It has been suggested in a previous study that a history of severe dysmenorrhea is associated with increased pain during CDBs [16], but in our study, dysmenorrhea was not significantly associated with biopsy pain scores based on linear regression analysis. From the multivariable analysis, we found that nulliparity and anxiety were significantly associated with increased biopsy pain scores. Previous studies noted that nulliparous women and women with anxiety might have a false impression that any procedures related to genital organs are painful [24,25,26]. Consequently, a 10% lidocaine spray may be used in nulliparous women and women with unpleasant experience and anxiety to decrease the adverse responses to CDBs.

***Research implication:*** Lidocaine spray is a simple method that could provide small pain reductions during CDBs. Nevertheless, such a reduction is small and probably is not clinically significant. Accordingly, because CDBs, though a minor procedure, can result in significant biopsy pain, as noted in this study, there is still a need for other more effective modalities. Future randomized controlled trials comparing other local cream or spray analgesics should be encouraged.

***Clinical implication:*** The application of lidocaine spray to the cervix has a benefit of reducing the pain associated with a CDB by a small amount, but routine use is not recommended. However, this method is safe and may be reasonably considered as an alternative in nulliparous and/or overly anxious women undergoing CDBs.

The strengths of this study are as follows: (1) The prospective randomized controlled design with an adequate sample size for the outcome of interest and allocation concealment. (2) All procedures were performed by the same level of gynecologic oncologists with the same technique. (3) All patients provided data for the final analysis of the main outcomes in the assigned groups without dropout. (4) There was a placebo group (normal saline solution spray), which showed that normal saline solution had no placebo effect in reducing pain during the procedure. The limitations of this study include the following: (1) The participant blinding process was only partial as those in the no-intervention group could not be perfectly blinded. (2) The blinding of the clinician could not be implemented in this situation. (3) Comparisons of the effectiveness between lidocaine spray and other spray analgesic or other local creams were not performed.

## 5. Conclusions

Since pain relief, even during a minor procedure like a CDB, which is commonly performed in daily gynecologic practice, is needed by most patients, and an accepted method for such a procedure is not well established, we conducted this study to evaluate the efficacy of lidocaine spray in reducing pain during CDBs. This study demonstrated that the application of lidocaine spray to the cervix has the benefit of reducing the pain associated with colposcopy-directed cervical biopsies by a small amount. However, the intervention is safe and may be reasonably considered in nulliparous and/or overly anxious women undergoing CDBs.

## Figures and Tables

**Table 1:** Participant characteristics.

Characteristic	Total (n = 312)	Lidocaine Spray (n = 104)	Normal Saline (n = 104)	Control Group (n = 104)	*p*-Value
Median age (range), years	41 (34–52)	42 (32–54)	40 (33–51)	42 (35–51)	0.74
Parity					
Nulliparous	77 (24.7)	23 (22.1)	28 (26.9)	26 (25.0)	0.74
Multiparous	235 (75.3)	81 (77.9)	76 (73.1)	78 (75.0)	
Menopause status					
No	228 (73.1)	77 (74.0)	75 (72.1)	76 (73.1)	0.99
Yes	84 (26.9)	27 (26.0)	29 (27.9)	28 (26.9)	
Dysmenorrhea					
No	182 (58.3)	60 (57.7)	58 (55.8)	64 (61.5)	0.72
Yes	130 (41.7)	44 (42.3)	46 (44.2)	40 (38.5)	
Dyspareunia					
No	239 (76.6)	78 (75)	78 (75)	83 (79.8)	0.67
Yes	73 (23.4)	26 (25)	26 (25)	21 (20.2)	
Anxiety					
No	103 (33)	32 (30.8)	37 (35.6)	34 (32.7)	0.78
Yes	209 (67)	72 (69.2)	67 (64.4)	70 (67.3)	
History of PID					
No	241 (77.2)	81 (77.9)	76 (73.1)	84 (80.8)	0.45
Yes	71 (22.8)	23 (22.1)	28 (26.9)	20 (19.2)	
HIV status					
No	288 (92.3)	97 (93.3)	95 (91.3)	96 (92.3)	0.96
Yes	24 (7.7)	7 (6.7)	9 (8.7)	8 (7.7)	
Cervical cytology result					
Negative/HPV+ve	30 (9.6)	7 (6.7)	13 (12.5)	10 (9.6)	
ASC-US	131 (42.0)	46 (44.2)	39 (37.5)	46 (44.2)	
ASC-H	6 (1.9)	2 (1.9)	2 (1.9)	2 (1.9)	0.83
LSIL	127 (40.7)	44 (42.3)	40 (38.5)	43 (41.3)	
HSIL	9 (2.9)	2 (1.9)	4 (3.8)	3 (2.9)	
AGC-NOS	5 (1.6)	2 (1.9)	3 (2.9)	0 (0.0)	
AGC-FN	2 (0.6)	1 (1.0)	1 (1.0)	0 (0.0)	
AIS	1 (0.3)	0 (0.0)	1 (1.0)	0 (0.0)	
Adenocarcinoma	1 (0.3)	0 (0.0)	1 (1.0)	0 (0.0)	
HPV status					
Negative	2 (0.6)	0 (0.0)	1 (1.0)	1 (1.0)	0.44
Positive	45 (14.4)	11 (10.6)	18 (17.3)	16 (15.4)	
Unknown	265 (84.9)	93 (89.4)	85 (81.7)	87 (83.7)	
Final histology					
Negative	76 (24.4)	26 (25.0)	29 (27.9)	21 (20.2)	
Chronic cervicitis	98 (31.4)	31 (29.8)	29 (27.9)	38 (36.5)	0.42
LSIL	109 (34.9)	38 (36.5)	38 (36.5)	33 (31.7)	
ASC-H	24 (7.7)	9 (8.7)	6 (5.8)	9 (8.7)	
HSIL	3 (1.0)	0 (0.0)	0 (0.0)	3 (2.9)	
AIS	1 (0.3)	0 (0.0)	1 (1.0)	0 (0.0)	
Adenocarcinoma	1 (0.3)	0 (0.0)	1 (1.0)	0 (0.0)	

Values are given as (n %), or median (range). AGC-NOSs, atypical glandular cells not otherwise specified; AIS, adenocarcinoma in situ; ASC-Hs, atypical squamous cells cannot exclude HSIL; ASCUS, atypical squamous cells of undetermined significance; HIV, human immunodeficiency virus; HPV, human papilloma virus; HSILs, high-grade squamous intraepithelial lesions; LSILs, low-grade squamous intraepithelial lesions; PID, pelvic inflammatory disease.

**Table 2:** Procedure information.

Characteristic	Total (n = 312)	Lidocaine Spray (n = 104)	Normal Saline (n = 104)	Control Group (n = 104)	*p*-Value
Procedure					
CDB	251 (80.4)	85 (81.7)	84 (80.8)	82 (78.8)	0.58
CDB + ECB	40 (12.8)	12 (11.5)	12 (11.5)	16 (15.4)	
CDB + ECC	7 (2.2)	1 (1.0)	4 (3.8)	2 (1.9)	
CDB + Polypectomy	9 (2.9)	5 (4.8)	1 (1.0)	3 (2.9)	
CDB + ECC + Endocel	5 (1.6)	1 (1.0)	3 (2.9)	1 (1.0)	
Number of biopsy					
2	291 (93.3)	96 (92.3)	94 (90.4)	101 (97.1)	0.13
3	21 (6.7)	8 (7.7)	10 (9.6)	3 (2.9)	
Median duration of colposcopy (minutes) (range)	8.0 (6.0–10.0)	10.0 (7.0–12.0)	9.0 (6.0–10.0)	7.0 (5.0–10.0)	<0.001 *
Transformation zone					
Type 1	173 (55.4)	60 (57.7)	54 (51.9)	59 (56.7)	0.76
Type 2	70 (22.4)	20 (19.2)	28 (26.9)	22 (21.2)	
Type 3	69 (22.1)	24 (23.1)	22 (21.2)	23 (22.1)	
Colposcopy diagnosis					0.28
Normal	15 (4.8)	8 (7.7)	5 (4.8)	2 (1.9)	
LSIL	262 (84.0)	83 (79.8)	86 (82.7)	93 (89.4)	
HSIL	35 (11.2)	13 (12.5)	13 (12.5)	9 (8.7)	

Values are given as n (%), or median (range). CDB, colposcopy-directed biopsy; ECB, endocervical brushing; ECC, endocervical curettage; HSILs, high-grade squamous intraepithelial lesions; LSILs, low-grade squamous intraepithelial lesions; Endocel, endometrial sampling with Endocel^®^ device. * statistically significant (*p* < 0.05).

**Table 3:** Kappa’s coefficient for agreement between self-sampling and clinician sampling.

Pain Score	Total (n = 312)	Lidocaine Spray (n = 104)	Normal Saline (n = 104)	Control Group (n = 104)	*p*-Value
Baseline	1.2 (0.2–3.1)	1.4 (0.3–3.5)	1.0 (0.2–2.7)	1.2 (0.3–3.1)	0.53
Biopsy	2.9 (1.2–5.3)	2.5 (1.2–4.9)	3.1 (1.2–5.6)	3.3 (1.3–5.3)	0.51
Biopsy to baseline change	1.3 (0.0–3.0)	0.9 (0.0–2.3)	1.8 (0.2–3.8)	1.4 (0.1–2.8)	0.03
Post-procedure	2.0 (0.7–4.1)	1.8 (0.5–3.1)	2.3 (0.8–4.8)	1.8 (0.8–4.0)	0.18
Post-procedure to baseline change	0.3 (−0.5–2.1)	0.1 (−0.8–1.8)	1.0 (−0.1–2.8)	0.3 (−0.7–1.9)	0.03

Values are presented as medians (interquartile range).

**Table 4:** Direct comparisons of the pain scores between study groups.

Variable	Median Difference	*p*-Values
Biopsy to baseline difference (scores)		
Lidocaine spray vs. normal saline	−0.9	<0.001 *
Lidocaine spray vs. control group	−0.5	0.06
Normal saline vs. control group	0.4	0.13
Post-procedure to baseline difference (scores)		
Lidocaine spray vs. normal saline	−0.9	0.01 *
Lidocaine spray vs. control group	−0.2	0.29
Normal saline vs. control group	0.7	0.03 *

Noted: All *p*-value perform by Dunn’s test for multiple comparisons; median difference [A vs. B] is median of A—median of B; * statistically significant (*p* < 0.05).

## Data Availability

The datasets analyzed during the current study are available from the corresponding author upon reasonable request.
